# Nanobacteria Are Mineralo Fetuin Complexes

**DOI:** 10.1371/journal.ppat.0040041

**Published:** 2008-02-15

**Authors:** Didier Raoult, Michel Drancourt, Saïd Azza, Claude Nappez, Régis Guieu, Jean-Marc Rolain, Patrick Fourquet, Bernard Campagna, Bernard La Scola, Jean-Louis Mege, Pascal Mansuelle, Eric Lechevalier, Yvon Berland, Jean-Pierre Gorvel, Patricia Renesto

**Affiliations:** 1 Unité des Rickettsies, Centre National de la Recherche Scientifique UMR 6020, IFR 48, Faculté de Médecine, Marseille, France; 2 Centre National de la Recherche Scientifique FRE2738, Faculté de Médecine Secteur Nord, Marseille, France; 3 Centre d'Immunologie de Marseille-Luminy, Aix Marseille Université, Faculté de Sciences de Luminy, Marseille, France; 4 INSERM, U631, Marseille, France; 5 Centre National de la Recherche Scientifique UMR 6102, Marseille, France; 6 Service d'Urologie, Hôpital de la Conception, Assistance Publique-Hôpitaux de Marseille, France; 7 Service de Nephrologie, Hôpital de la Conception, Assistance Publique-Hôpitaux de Marseille, France; The Salk Institute for Biological Studies, United States of America

## Abstract

“Nanobacteria” are nanometer-scale spherical and ovoid particles which have spurred one of the biggest controversies in modern microbiology. Their biological nature has been severely challenged by both geologists and microbiologists, with opinions ranging from considering them crystal structures to new life forms. Although the nature of these autonomously replicating particles is still under debate, their role in several calcification-related diseases has been reported. In order to gain better insights on this calciferous agent, we performed a large-scale project, including the analysis of “nanobacteria” susceptibility to physical and chemical compounds as well as the comprehensive nucleotide, biochemical, proteomic, and antigenic analysis of these particles. Our results definitively ruled out the existence of “nanobacteria” as living organisms and pointed out the paradoxical role of fetuin (an anti-mineralization protein) in the formation of these self-propagating mineral complexes which we propose to call “nanons.” The presence of fetuin within renal calculi was also evidenced, suggesting its role as a hydroxyapatite nucleating factor.

## Author Summary

In the last decade, the exact nature of nanobacteria was one of the most controversial of scientific questions. An audacious theory proposed the existence of nanobacteria, initially discovered in Italian hot spring deposits, as a new life form responsible for a wide range of diseases in humans, thus qualifying them as new agents of emerging infectious diseases. The community of microbiologists remained therefore skeptical about the fact that such structures, 100 times smaller than bacteria and highly resistant to heat and other treatments that would normally kill the latter, could be living entities fully capable of self-replication. Other scientists wondered if they might be an unusual form of crystal rather than micro-organisms. The comprehensive characterization of nanobacteria was the focus of our study. Our results definitively ruled out the existence of nanobacteria as living entities and revealed that they correspond to self-propagating mineral-fetuin complexes that we called “nanons.”

## Introduction

“Nanobacteria” are mysterious particles that have spurred one of the biggest controversies in modern microbiology [1–3]. First discovered by geologists as 100 nm coccoid particles present on mineral surfaces [4], such structures were later found in human and cow blood as well as in commercial cell culture serum [5]. The culturability of “nanobacteria” was then reported by Kajander's team [6] who established a link between these particles and kidney stone formation [7]. The data described by Cisar's group reached completely opposite conclusions as Kajander's original assertion considering nanobacteria as living microorganisms [2]. In contrast to what would be expected from growth of a living entity, Cisar et al. failed to detect nucleic acids and suggested that observed biomineralization may be initiated by non living macromolecules generating self propagating microcrystalline apatite. In the last few years, these calcifying nanoparticles have been associated with several human diseases including polycystic kidney disease, renal calculi, and chronic prostatitis [8]. However, despite the various pathological disorders they cause, whether nanobacteria are living or nonliving cells is still under debate [9].

Here, a comprehensive analysis was undertaken with the Nanobacterium sp. strain Seralab 901045 provided by O. Kajander (Nanobac Oy, Kuopio, Finland), in order to gain better insights on such a propagating calcifying agent putatively endowed with pathogenic properties. To address this question, several features of the “nanobacteria” previously reported were examined in detail including their propagation conditions, susceptibility to various chemical and physical treatments and their effect on eukaryotic cell viability. Their nucleic acid and proteomic content was also carefully analyzed. The antigenic properties of “nanobacteria” were investigated by immunization of mice with such particles and the specificity of the response was determined against bacteria from *Rickettsia*, *Coxiella* and *Bartonella* genera.

Our data provided evidence that the particles previously attributed to “nanobacteria” are self-propagating mineral-fetuin complexes that we propose to call “nanons.”

## Results

### Growth of Nanons on Axenic Media

After a 10-d incubation in DMEM supplemented with 10% heat-decomplemented fetal calf serum, nanons formed a clearly visible film at the bottom of culture flask. Subculturing was done every 14 d at a 1:10 dilution. Also, serum-free DMEM sustained both growth and subculture of nanons following the same procedure. Under these conditions, the ability to form particles was transferable for 3 to 5 passages. In contrast, nanons did not grow in vitamin-free DMEM. However, addition of any one of the 11 vitamins comprised in this medium restored growth. Growth on Loeffler medium was not successful.

### Analysis of Nucleotidic Content of Nanons

Hoechst 33342 did not stain nanons. In contrast, small fluorescent particles were visible after acridine orange and DAPI staining. Among the 22 PCR assays targeting 16S rRNA gene, 8 yielded amplicons. Their DNA sequence was found to be 99% similar to that of Arthrobacter luteolus in 2 cases and 97% similar to that of Bacillus sp. in other ones. Two amplicons exhibited similarities with Stenotrophomonas maltophila (99%) and Pseudomonas sp. (99%), respectively. Mixed sequences were obtained in the remaining 2 cases. All attempts to extract RNA failed (not shown).

### Susceptibility of Nanons to Various Chemical and Physical Agents

Exposure of nanons to DNAse (10 μg/mL) or RNAse (16 μg/mL) did not alter particle formation. In contrast, we observed that nanon propagation was suppressed following UV or 32 Ggy gamma ray irradiation. The nanon monolayer was also destroyed after a 3- to 5-s contact with either trypsin (0.5%) or EDTA (50 mM) as well as under acidic conditions (DMEM pH 5), as shown in real-time movies (http://ifr48.timone.univ-mrs.fr/files/Nanons_Videos/Nanons_films_14.06.07.zip). In contrast, alkalization of the buffer in the 8 to 10 range and exposure to proteinase K (100 μg/mL) failed to alter the growth.

We also investigated the effect of some antibiotics including gentamicine, cotrimoxazole, doxycycline and oxytetracycline hydrochloride. While a subtle decrease in the number of particles counted at day 10 was noted for the last two compounds, we failed to observe a significant inhibition of nanon propagation as compared to the control.

### Staining, Morphological Analysis, and Antigenicity of Nanons

**Figure 1:**
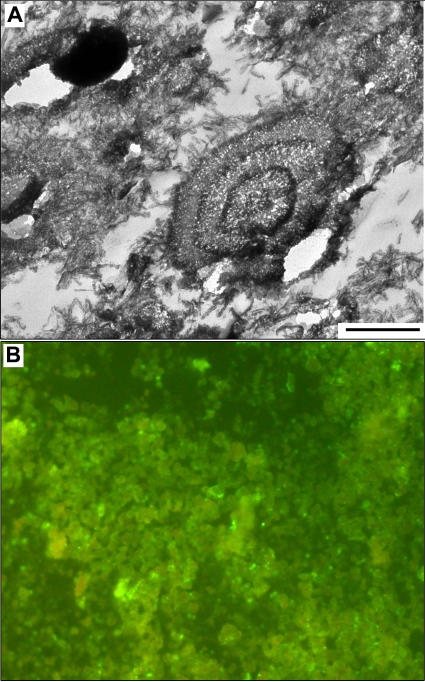
Morphological Analysis of Nanons (A) Transmission electron microscopy of nanons featuring a dense core surrounded by a loose halo (bar = 500 nm). (B) Immunofluorescence staining of nanons with anti-nanon antibodies (1:160). Magnification ×100.

Nanons did not stain with Gimenez or Gram stains. When observed by electron microscopy, they were visualized by a central core, a first ring and a second electron-dense ring (Figure 1A). Scanning microscopy indicates a double population, including a first population with Gaussian repartition and a 431 ± 10 nm size with larger nanons appearing twice as large as budding nanons.

These particles exhibited antigenic properties. Thus, sera of mice immunized with nanons exhibited a positive signal when tested by immunofluorescence while any recognition was detected with pre-immune sera (Figure 1B). These antibodies were specific since they failed to recognize microorganisms from the genus *Rickettsia*, *Bartonella* and *Coxiella* when tested at the same dilution (not shown).

### Effect of Nanons on Eukaryotic Cell Viability

Nanons had a deleterious effect on amoebae. Thus, when these eukaryotic cells were cultivated in the presence of nanons, we observed less living trophozoïtes (*p* = 0.008, Kruskal-Wallis test) and cysts (*p* = 0.001, Anova test). The number of dead trophozoïtes increased (*p* = 0.05, Kruskal-Wallis test) in comparison with nanon-free amoebae. However, the major difference was the larger number of dead cysts observed in amoebae cultivated with nanons (*p* < 0.0001). Nanons also had a deleterious effect on THP-1 and HeLa cells. In the presence of 10% fetal calf serum, nanons did not affect the viability of each cell type. In the presence of 2% fetal calf serum, nanons increased cell death after 24 h in both THP-1 cells (4% ± 1% vs 12% ± 2%, *p* < 0.002) and HeLa cells (15% ± 3% vs 30% ± 5%, *p* < 0.001). Beyond 24 h, nanons did not modify the survival pattern of THP-1 cells but they amplified cell death in HeLa cells (22% ± 3% vs 58% ± 9%, *p* < 0.0001 after 3 d).

### Evidence for the Presence of Proteins in Nanon Extracts

**Figure 2:**
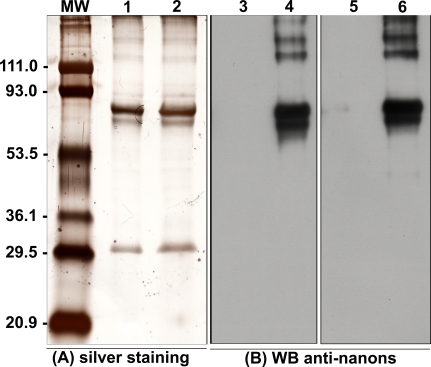
Silver-Stained SDS-PAGE and Western Blot Analysis of Nanon Proteins (A) Silver staining of 2.5 μg (lane 1) or 5 μg (lane 2) of nanon extract subjected to 10% SDS-PAGE. (B) Western blot performed with two distinct mouse anti-nanon antibodies (1:2,500). Lanes 3 and 5, pre-immune sera; lane 4, mice 1; lane 6, mice 2. Molecular weight markers (MW) are on the left side.

Analysis of nanons by SDS-PAGE after mineral phase dissolution with EDTA revealed that proteins were present in our sample (Figure 2A). Three major bands with an apparent MW of 70, 65 and 30 kDa were observed. This profile was close to that reported for biofilms-associated macromolecules generated from saliva samples [2]. Here, the DMEM medium used for propagation was not supplemented with bovine serum and was certified free of proteic contaminants (Gibco-BRL). Accordingly, when the same experiment was performed using DMEM concentrated by the lyophilization process, we failed to observe these bands (not shown). A Western blot analysis was then performed using sera of immunized mice. Results obtained (Figure 2B*)* showed that both the 70- and 65-kDa bands were antigenically positive when probed with anti-nanon antibodies in contrast with the 30-kDa product. These results suggested that corresponding proteins were specific from the nanon particles.

### Evidence for the Presence of Fetuin in Nanons

**Figure 3:**
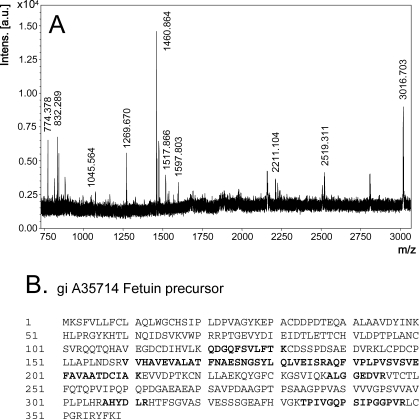
Identification of Nanon Proteins by a Proteomic Approach (A) MALDI-TOF chromatogram obtained from the tryptic digest of the 65-kDa protein excised from silver-stained gel. (B) Mass and sequences of tryptic peptides mapping to the identified sequence are underlined. This spectrum is representative for 2 distinct experiments.

In order to identify the proteins present in nanon samples, different strategies were applied. First, a MALDI-TOF mass spectrometry analysis was carried out on the bands excised from an SDS-PAGE silver-stained gel. The mass spectrum shown in Figure 3A corresponds to the trypsin digest of the protein with an observed MW of 65 kDa. Only peptide masses obtained from this spectrum and differing by less than 0.1 Da than those determined by a virtual tryptic digestion of the candidate proteins were considered. Using such parameters, the highest identification score corresponded to the bovine fetuin precursor (gi_A35714). As illustrated in Figure 3B, 27% coverage of this theoretical protein sequence was obtained. This result was confirmed by direct N-terminal sequencing which was attempted in parallel and allowed identification of 12 residues of the primary protein sequence which are QPLDPVAGYKET. With the exception of the first and the last amino acid residue, this peptide sequence shares 100% homology with the previously identified fetuin. This proteomic approach also led to the identification of the 30-kDa protein as the apolipoprotein A-1 precursor (coverage 53%) which has a predicted molecular weight of 30.2 kDa.

### Confirmation of the Fetuin Presence within Nanons

**Figure 4:**
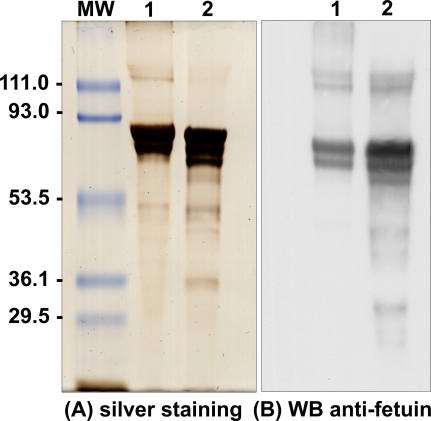
Comparative SDS-PAGE and Western Blot Analysis of Nanon Proteins and Fetuin Nanon proteins and bovine fetuin resolved by 10% SDS-PAGE were visualized by silver staining (A) or transferred to nitrocellulose before probing with the mouse anti-bovine fetuin antibodies (1:10,000) (B). Lane 1, nanons (5 μg); lane 2, bovine fetuin (5 μg). Molecular weight markers (MW) are indicated on the left.

**Figure 5:**
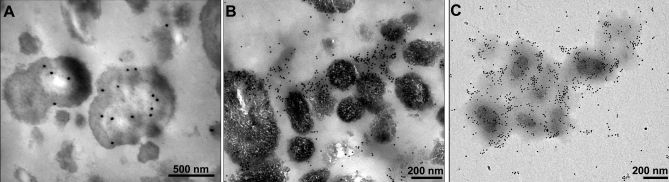
EM Analysis of Nanons Following Immunogold Staining (A) Post-embedding staining of nanons with either the anti-nanon antibodies (1:200, bar 500 nm) or (B) with the anti-fetuin antibodies (1:400, bar 200 nm). (C) Whole mount preparation of nanons stained with anti-fetuin antibodies (1:400, bar 200 nm).

Interestingly, we observed a close pattern for both nanon extracts and commercially available fetuin following silver staining (Figure 4A). On a 10% SDS-PAGE, the latter has an apparent molecular weight higher than that expected and several bands ranging from 64 to 70 kDa are observed (line 2, Figure 4A). The presence of fetuin in nanons was next examined by Western blotting. As shown in Figure 4B both nanons and purified fetuin were recognized by anti-fetuin antibodies. A similar profile was observed with anti-nanon antibodies. In contrast, the monoclonal anti-nanobacteria antibodies (NanoVision 8D10, Nanobac, Kuopio, Finland) gave a less specific pattern (Protocol S1 and Figure S1). Post- and pre-embedding immunogold staining of nanons indicated that fetuin was located both inside the particles and at their surface (Figure 5).

The amount of alpha-fetuin, estimated through an ELISA-based approach, ranged from 31–227 pg in the whole nanon particules to 44–117 pg in the corresponding serum-free DMEM culture supernatants. Similarly, the amount of total proteins, glucose and of several minerals including sodium, potassium, chlorine and calcium were found identical in both fractions. In contrast, other biochemical analyses indicated that nanon particles were depleted in phosphorus (0.7 mmol/L versus 3.6 mmol/L) and CO_2_ (6 mmol/L versus 23 mmol/L) in comparison with supernatants.

### Recognition of Kidney Stone Proteins by Anti-Nanon and Anti-Human Fetuin Antibodies

**Figure 6:**
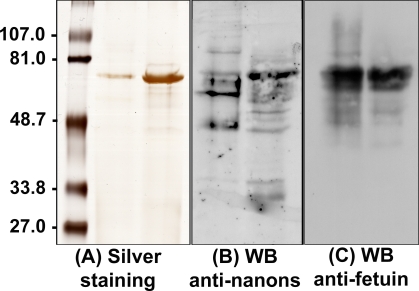
SDS-PAGE and Western Blot Analysis of Proteins Extracted from Human Kidney Stones 50 μl of proteic extracts obtained from human kidney stones of 2 distinct patients were subjected to 10% SDS-PAGE and silver-stained (A). Alternatively, obtained gels were transferred on a nitrocellulose membrane before probing with anti-nanon ([B], 1:2,500) or anti-human fetuin ([C], 1:10,000) antibodies. Molecular weight markers (MW) are on the left side.

The soluble material obtained from human kidney stone extracts showed well-defined bands when analyzed by SDS-PAGE (Figure 6). Western blot analysis demonstrated that the major soluble component present in such samples is recognized by the anti-nanon antibodies. A strong pattern of recognition was also obtained with anti-human fetuin antibodies. Accordingly, the fetuin concentration measured in 5 specimens ranged from <0.1 pg/ml to 0.84 pg/ml.

## Discussion

### Nanon Is Not a Microorganism

Consistent with data published by Cisar et al. [2], we failed to clearly demonstrate the presence of nucleic acids in nanons. Indeed, we observed discrepant results using various nucleic acid stains, such as nanons being easily stained by orange acridine but poorly stained by DAPI and Hoechst 33342. Also, the growth of nanons was not altered in presence of either DNAse or RNAse. Finally, 16S rRNA gene amplification and sequencing most often identified α-proteobacteria and γ-proteobacteria, both known to be waterborne contaminants in PCR-based experiments [10]. 16S rRNA gene sequence of Nanobacterium sanguineum (GenBank accession number X98418) and Nanobacterium sp. (GenBank accession number X98419) have been previously found to be indistinguishable from those of Phyllobacterium mysinecearum, a microorganism identified as a source of contaminating 16S rDNA in PCR studies [2]. It is thought that previously reported 16S DNA amplifications by PCR using “nanobacteria” as template result from PCR artifacts [2,11]. These data led us to hypothesize that nanons might have the ability to trap any contaminant 16S rDNA fragment present in the medium or environment rather than displaying original sequences from an emerging microorganism. All together, the data suggest that the nanon is a nucleic-acid free, transferable biological entity.

### Nucleation of Apatite Involves a Proteic Component

The spectacular dissolution of nanons exposed to the calcium chelator EDTA or to acidic medium confirmed that the formation of these particles is related to a calcification process [1]. As previously mentioned [2], such a demineralization could explain the inhibition of nanon growth observed with some antibiotics [12]. When tested at concentrations comparable with the prescription in human beings [13], we failed to detect a significant inhibitory effect of antibiotics against nanon growth.

Apatite is the major constituent of nanons but these particles are also composed of other unidentified compounds. When injected into mice, nanons induced a specific immune response. This is consistent with a recent report about a scientist who was accidentally exposed to a splash of nanobacteria in the eye in 1993 and who still exhibits a high IgG titer against these particles [14]. Such antigenic feature cannot be related to apatite. Indeed, the non-immunogenic nature of this biomineral supports its use for mineralizing applications in preventive and restorative dentistry. The existence of apatite-protein complexes within nanons was related a few years ago but the nanon-associated proteins were not characterized [15]. The putative role of one or several proteins in nanon propagation is also supported by the inhibitory effects of gamma or UV irradiation. Indeed, exposure of proteic molecules to these treatments can provoke secondary conformational changes of proteins which alter their functional properties in turn [16]. This hypothesis was emphasized by the inhibitory activity of trypsin evidenced here for the first time.

### Nanon Are Fetuin-Mineral Complexes

SDS-PAGE analysis of nanons was achieved from samples grown in DMEM without serum for at least 2 passages. This procedure allowed discarding of any adsorption of seric proteins to apatite. The profile of silver-stained proteins was found similar to that reported by Cisar et al., who worked on “nanobacteria” originating from saliva [2]. Based on the discrepancy between the low number of proteins detected and that expected from a putative microbial proteome, these proteins were considered as salivary contaminants. We then found that the 65-kDa protein was recognized by anti-nanon antibodies. Interestingly, recent experiments aimed to measure ^35^S-methionine incorporation in nanon cultures showed that one protein with similar size was primarily labeled during the course of replication [17]. By using two different approaches used here, this protein was identified as fetuin. When commercially available purified fetuin was analysed by SDS-PAGE, several bands were visualized. This migration profile appeared closely related to that in nanons. The observed MW of the largest protein was higher than the theoretical fetuin MW of 48 kDa. Such a shift in migration was previously described and most probably results from glycosylations [18]. Fetuin (α2-Heremans Schmid glycoprotein) is indeed a plasma protein exhibiting several secondary modifications including *N*- and *O*-glycosylations. Such post-translational modifications could also afford the recognition of nanons by chlamydial LPS antibodies [19]. The presence of fetuin within nanons was further confirmed by a Western blot and immunogold staining. In addition, we noticed that purified fetuin was recognized by anti-nanon antibodies.

### Presence of Fetuin in Human Calculi

The recovery of nanons from human calculi and the observation of nanoforms in calcified arterial tissues [8] are compatible with the presence of fetuin in such samples. Accordingly, this protein was observed in calcified vascular smooth muscle cells [20]. Several studies aimed at identifying renal stone proteins hypothesized to play a role in stone formation were achieved [21]. Here, we provided evidence for the first time for fetuin association with renal stones.

### Concluding Remarks

As recently reviewed [9], while associated with several diseases, nothing was done to identify or characterize the novel form of life known as “nanobacteria.” Here, we demonstrated these particles are self-propagated mineral protein complex containing fetuin as the major biological component which we propose to call nanons.

The serum protein fetuin was described as a potent inhibitor of apatite formation and of calcium phosphate mineral precipitation [22]. Its inhibitory activity was shown to be mediated by the transient formation of a fetuin-mineral complex [23] also described as colloidal calciprotein particles containing fetuin, calcium and phosphate [24]. In fact, the comparison of the electron microscopy structure of these 30- to 150-nm particles [24] to that of nanons indicated that these particles are closely related. This relationship was never pointed out before. Recently, a strong correlation between the serum levels of the fetuin-mineral complex and arterial calcifications induced by vitamin D in a rat was demonstrated, supporting the blood-borne theory of artery calcification [25]. However, the biochemical basis of these findings, which are somewhere in contradiction with the inhibitory known effect of fetuin against mineralization, were not clarified. The propagation of nanons in vitro suggests that fetuin should promote hydroxyapatite nucleation. Accordingly, it was demonstrated that polyanionic proteins can nucleate crystal formation when adsorbed onto a rigid substrate while they exert an inhibitory effect when free in solution [26]. We can also hypothesize that the conformational change of the fetuin protein, equivalent to that observed in prions, can occur [27]. This highly speculative hypothesis leads to a new “pathogenic” fetuin isoform able to induce hydroxyapatite crystallization and to promote calcification. In this respect, we noticed that the prion preparations are often contaminated by nucleic acids [28] as observed for nanons. It will be highly interesting in future studies to gain a greater understanding of the mechanisms by which fetuin promote mineralization. This should be helpful to design future therapeutic strategies for the treatment of kidney stones and other pathological states to which “nanobacteria” have been associated [8].

## Materials and Methods

### Growth and culture medium.

Nanobacterium sp. strain Seralab 901045 (herein designed as “nanons”) provided by O. Kajander (Nanobac Oy, Kuopio, Finland) was propagated in DMEM (Invitrogen, Paisley, UK) with 10% heat-decomplemented fetal calf serum (Gibco-BRL, Invitrogen, Paisley, UK) in sterile plastic culture flasks (Becton Dickinson, Le Pont de Claix, France) at 37°C under room atmosphere. Subculture was done every 14 d by doing a 1:10 dilution in this complete culture medium. Growth was then attempted in Loeffler medium (BioMérieux, Marcy-l'Etoile, France) and in fetal calf serum-free DMEM. Determination of a minimal culture medium was assayed by subculturing nanons into a chemically defined medium obtained by stepwise addition of every one of the DMEM constituents to sterile distilled water up to obtaining the whole medium reconstitution.

### 16S rRNA gene amplification and sequencing.

The 16S rRNA gene was amplified by PCR using nanon-extracted DNA as a template (QIAamp tissue kit, QIAGEN, Hilden, Germany) and with universal primers fD1 and rp2 [29] (Eurogentec, Seraing, Belgium). Sequencing of amplified products was carried out as previously described [30]. The resulting sequences were compared with those available in the GenBank database using the gapped BLASTN 2.0.5 program through the National Center for Biotechnology Information server.

The GenBank accession numbers for the sequences determined in this work were EF585587 to EF585591.

### Susceptibility to physical agents.

The growth of nanons was studied in serum-free DMEM after UV irradiation and 35 Ggy gamma-irradiation (Isotron, Marseille, France). In these experiments, nanon growth was detected by weekly observation of a white film at the bottom of the flask and by a smear preparation of acridine orange staining. Every experiment was done three times.

### Susceptibility to chemical agents and under various pH.

The growth of nanons was studied in serum-free DMEM after a 60 min incubation at 37°C in the presence of either 50 μg/mL DNAse (Invitrogen), 50 μg/mL RNAse (Roche Diagnostics, Mannheim, Germany), 50 mM EDTA (Sigma-Aldrich, Saint-Quentin Fallavier, France), 0.5% trypsin (Gibco-BRL) or 100 μg/mL proteinase K (Eurobio, Les Ulis, France). Growth was studied in parallel after heat-inactivation (10 min at 70°C) of the above-mentioned enzymes. Enzyme-treated nanons were plated onto a 150 cm^2^ culture flask and incubated for 4 wk at 37°C in the presence of serum-free DMEM. Growth of nanons was detected by the weekly observation of a white film on the bottom of the flask and by a smear preparation of acridine orange staining. Growth of nanons at various pH was determined in sterile 96-well microtitration plates. The wells were filled with 100 μl of nanons resuspended in serum-free DMEM (10^6^ particles/mL) and the microtitration plate was maintained at 37°C for 10 d, i.e., up to the formation of a visible white film at the bottom of each well. The pH of both the serum-free DMEM and of the nanon culture supernatant was measured. This supernatant was replaced by fresh DMEM supplemented with the appropriate volume of sterile HCl or NaOH in order to yield DMEM ranging from pH 4 to 10. Wells were observed daily for the presence of a white film and nanons were observed by acridine orange staining of a cytospin smear and counted by using the Microcyte cell flow cytometer (CellD, Roquemaure, France) after a 5-d incubation at 37°C. Each condition was tested three times.

### Antibiotic susceptibility.

Antibiotics used in this study were doxycycline (Pfizer, Neuilly, France) at 10 μg/ml, oxytetracycline hydrochloride (Sigma-Aldrich) at 10 μg/ml, gentamicin (Dakota Pharm, Creteil, France) at 10 μg/ml and cotrimoxazole (Roche Diagnostics) at 80 μg/ml of sulfamethoxazole. Growth of nanons was performed for 10 d as described above before being harvested and diluted 1:20 in DMEM. Diluted nanons were subcultured in a 25 cm^2^ culture flask supplemented or not with antibiotics. For each condition, nanons were numerated by using the Microcyte apparatus at day 0 and after 3, 7 and 10 d of incubation at 37°C to monitor the kinetic of growth. Experiments were carried out twice.

### Cell co-cultures.

*Cells:* THP-1 monocytic cells and HeLa cells were cultured in RPMI 1640 containing 2% or 10% fetal calf serum, 25 mM HEPES, 2 mM L-glutamine, 100 U/ml penicillin, and 100 μg/ml streptomycin (Invitrogen). THP1 cells and HeLa cells (5 × 10^5^ cells/ml) were grown in presence of nanons for different periods. After washing to remove unbound nanons, HeLa cells were incubated with 500 μL trypsin/EDTA for 2 min. The viability of suspended THP1 and Hela cells was determined by trypan blue dye test exclusion. Results are given as the mean ± SE. Statistical analysis was conducted with analysis of variance and regression analysis. Differences were considered significant when *p* < 0.05.

*Amoeba:* In amoeba co-culture experiments, 2 wells containing 2 mL of a suspension of Acanthamoeba polyphaga amoebae (10^5^ cell/ml) in Page's Amoebal Saline (PAS), prepared as described [31], were inoculated with 100 μL of a suspension of nanons in the same medium. Four control wells inoculated with 100 μl of PAS were used as controls. Viability of trophozoites and kysts were determined by trypan blue exclusion for 10 d.

### Morphology and electron microscopy.

Nanons were tentatively stained by using Gimenez, Gram and orange acridine staining, as well as with two DNA dyes, namely Hoechst 33342 and DAPI (Molecular Probes, Eugene, Oregon). For transmission electron microscopy, nanons were pelleted by centrifugation and prefixed for 1 h at room temperature in 2.5% glutaraldehyde (Electron Microscopy Sciences, Hatfield, PA, USA) in phosphate buffer saline (PBS), pH 7.2. After a 1-h washing in PBS, they were fixed for 1 h at room temperature with 1% osmium tetroxide, dehydrated through increasing concentrations (25% to 100%) of ethanol, and embedded in Epon 812 resin (Electron Microscopy Sciences). Thin sections were cut and poststained with a saturated solution of methanol-uranyl acetate and lead citrate in water before examination on a Philips Morgagni 268 D electron microscope (FEI Compagny France, Limeil-Brevannes, France).

### Production of polyclonal antibodies.

*Polyclonal anti-nanon antibodies:* Two 6-wk-old female Balb/C mice were inoculated intraperitoneally with 20 μg of nanons emulsified in Freund's complete adjuvant (1:1, v/v). Two booster doses were given in Freund's incomplete adjuvant at 14-d intervals. Bleeding was performed 2 wk after the last immunisation under Forene (Abbott, Rungis, France) anaesthesia and serum separated by centrifugation was stored at 4°C until use.

*Polyclonal anti-bovine fetuin antibodies:* One 6-wk-old female Balb/C mouse was inoculated intraperitoneally with 10 μg bovin fetuin (Sigma-Aldrich) mixed with 400 μg aluminium hydroxide and 10 μg CpG as previously described [32]. Following two booster doses at 14-d intervals, bleeding was performed as described above.

### Immunofluorescence assays.

For immunofluorescence assays, nanons were deposited on slides with a pen nib then air dried and fixed in acetone for 2 min. Wells were saturated by a 30-min incubation with PBS supplemented with 5% bovine serum albumin (BSA) and overlaid with 30 μl serum dilutions in PBS-BSA 3%. Bound antibodies were detected with a goat F(ab′)_2_ fragment anti-mouse IgG-FITC conjugated (Beckman-Coulter, Marseille, France) diluted 1:400 in PBS-BSA 3% containing 0.2% Evans blue (Bio-Mérieux, Marcy l'Etoile, France). All incubations were performed for 30 min in a moist chamber at 37°C and were followed by a washing in PBS two times of 10 min each, 5 min rinsing in demineralised water and drying in air. Slides were mounted with fluoprep (Bio Mérieux) then examined under an Olympus BX51 epifluorescence microscope at ×400 magnification. Sera of healthy mice were used as negative controls.

### Preparation of nanon samples for SDS-PAGE migration.

Two flasks (175 cm^2^) containing nanons grown in DMEM without serum supplementation were scraped and the resulting suspension was centrifuged (10,000*g*, 30 min, 4°C). The pellet was resuspended in 300 μl EDTA 1 M, pH 8.9 and dialysed for 5 h against 2 l Tris-HCl 50 mM, pH 8.0, EDTA 2 mM and using Spectra/Por 2 Dialysis Membranes (12–14 kDa molecular weight cutoff**,** Spectrum Laboratories, CA, USA). Final protein concentration of the sample was determined using a BioRad Protein Assay (Hercules, CA, USA) and estimated to be around 50 μg/flask.

### SDS-PAGE and Western blotting.

Nanons or bovine fetuin were fractionated on 10% polyacrylamide gels before silver staining [33]. Alternatively, separated proteins were transferred onto a nitrocellulose membrane (Trans-blot Transfer Medium, Biorad, Hercules, CA, USA) at 100 V for 1 h. Following a 1-h incubation with 5 % non-fat dry milk in PBS-Tween 20 (0.2%) as the blocking reagent, membranes were probed for 1 h with mice polyclonal anti-nanon antibodies or anti-bovine fetuin antibodies. Membranes were then washed 3 times for 10 min in PBS-Tween and incubated for an additional hour with horseradish peroxidase-conjugated goat anti-mouse secondary antibodies (Amersham Biosciences, 1:1,000). After washings, blots were revealed by chemiluminescence assays (ECL, Amersham Biosciences). The resulting signal was detected on Hyperfilm ECL (Amersham Biosciences) by using an automated film processor (Hyperprocessor, Amersham Biosciences).

### Analysis of nanon proteins by mass spectrometry.

Major bands observed on silver-stained SDS-PAGE were excised from the gels and subjected to in-gel digestion with trypsin (Sequencing grade modified porcine trypsine; Promega, Madison, WI, USA) as described [33]. Resulting tryptic peptides were then analysed by a matrix-assisted laser desorption ionization-time of flight (MALDI-TOF) mass spectrometer (Bruker Ultraflex spectrometer, Bruker Daltonics, Wissembourg, France) previously calibrated using autolytic peptides from trypsin. The peptide mass data obtained were analysed using Mascot software available online (http://www.expasy.org/tools/peptident.html). In some experiments, separated proteins were transferred onto an Immobilon-P PVDF-membrane (Millipore, Bedford, MA, USA) in semi-dry blotter equipment (Hoefer TE 77, Amersham Biosciences) for 2 h at a constant current of 400 mA, 80 W. Major bands visualised by staining with 0.02% Ponceau S red in 1% acetic acid were excised and stored in methanol for subsequent N amino acid sequence analysis by automated Edman degradation carried out using a Model 476A pulsed liquid protein sequencer (Applied Biosystems).

### Chemical and proteic composition of nanons and of renal calculi.

The concentrations of sodium, potassium, chlorine, CO_2_, total proteins, glucose, calcium, and phosphorus were measured in parallel into serum-free DMEM nanon supernatant and nanon particles by using the Beckman LX apparatus for biochemical analyses in clinical biochemistry. Before assays, culture supernatants and nanons were lyophilized and resuspended into 1 ml of DMEM or distilled water. The concentration of alpha-fetuin was determined from pellets and supernatants of 5 distinct nanon cultures by using a home-developed enzyme-linked immunosorbent assay (ELISA) technique. The concentration of alpha-fetuin was also estimated from the renal calculi weighed and then ground using ultrasonic sounds and mixed with 500 μL of Tween 20. The ELISA test was carried out on the supernatant obtained after centrifugation (800*g*, 10 min). Alpha-fetuin polyclonal antibodies, streptavidine-coupled antibodies and recombinant alpha-fetuin used in this assay were from R&D Systems Europe (Lille, France). The optical density was measured at 450 nm (EMS reader) procedure. The limit of detection was of 0.1 pg.

### Immunogold labeling.

The post-embedding immunogold labeling of nanons was done following a method previously described [34] with some modifications. Briefly, nanon pellets were fixed overnight in 2% glutaraldehyde and were then treated with 1% (v/v) osmium tetroxyde before dehydratation through an ethanol series and infiltration with EPON 812 resin. Polymerization was for 72 h at 60°C. Ultrathin sections (70 nm) were cut using an Ultramicrotome (ULTRACUT E Leica), collected on grids without formvar (Electron Microscopy Sciences) and then incubated in PBS buffer supplemented with 0.2% BSA (Roche Diagnostics). The sections were then treated with 0.05 M lysine (Sigma-Aldrich) in order to neutralize putative residual aldehyde groups. After repeated washings with PBS, the sections were then incubated for 3 h at 37°C in a humidified atmosphere with mouse anti-nanon antibodies (1:200) or anti-fetuin antibodies (1:100). The secondary reaction used goat anti-mouse antibodies conjugated to 25 nm colloidal gold particles (Aurion EM Reagents, the Netherlands) followed by washings with PBS and H_2_O. Gold particles were visualized using the R-GENT SE-EM silver enhancement kit (Aurion), following the instructions of the manufacturer. Labeled sections were viewed with a Philips electron microscope (MORGAGNI 268D) at 80 kV.

Whole-mount immunogold staining was carried out on nanons deposited on 400 mesh formvar coated Cu-grids (Electron Microscopy Sciences), fixed with 1% glutaraldehyde and treated with 50 mM NH_4_Cl. Following a preincubation step in a solution containing 1% normal goat serum (NGS, Aurion), 1% BSA and 0.2% Tween-20, nanons were labeled by sequential incubations with the mouse anti-fetuin antibodies (1:400) followed by reaction with a secondary goat-anti-mouse antibody conjugated with 10 nm colloidal gold particles (Aurion). After washings and an aldehyde-fixation, a treatment with 1% phosphotungstic acid pH 7.2 (ICN Biomedicals) was done to produce an intense electron-opaque stain and samples were viewed as described above.

### Western blot analysis of human kidney stones.

Five human kidney stones were suspended in 100 μl EGTA 0.5 M, pH 9.0 and mechanically broken by sonification. Each sample was then diluted in half in Laemmli buffer and heated 5 min at 95°C. The unbroken stone pieces were removed by centrifugation (10,000*g*, 10 min) and corresponding supernatants were processed for immunoblot analyses using either anti-nanon or anti-fetuin antibodies produced in the laboratory.

## Supporting Information

Figure S1 Western Blot Recognition of Nanons with Monoclonal Antibodies against Nanobacteria (NanoVision 8D10) (A) Commassie brilliant blue staining of nanon extract (5 μg) subjected to 10% SDS-PAGE.
(B) Western blot performed with polyclonal anti-nanon antibodies (1:1,1000), polyclonal anti-fetuin antibodies (1:10,000) or monoclonal anti-nanobacteria antibodies (NanoVision 8D10, Nanobac; 100 μg/ml). The molecular weight markers (MW) are on the left.
(19.3 MB TIF)

Protocol S1 Western Blotting of Nanons with Various Antibodies (21 KB DOC)
