# Supplementary material for: Nanobacteria Are Mineralo Fetuin Complexes
Source: PLoS Pathog. 2008 Feb 15;4(2):e41. doi: 10.1371/journal.ppat.0040041 (PMC2242841; doi:10.1371/journal.ppat.0040041)
Supplement: Supplementary file 1 [file ppat.0040041.sd001.doc]

**Protocol S1.** Nanons separated on 10% polyacrylamide gels (5 µg/well) were stained with

Coomassie brilliant blue dye or transferred onto a nitrocellulose membrane (Trans-blot Transfer Medium, Biorad, Hercules, CA, USA) at 100 V for 1 hour. Following a 1 hour incubation with 5 % non-fat dry milk in PBS-Tween 20 (0.2%) as the blocking reagent, membranes were probed for 1 hour with mice polyclonal anti-nanon antibodies, polyclonal anti-bovine fetuin antibodies or monoclonal anti-nanobacteria antibodies (NanoVision™ 8D10, Nanobac, Kuopio, Finland). Membranes were then washed 3 times for 10 min in PBS-Tween and incubated for an additional hour with horseradish peroxidase-conjugated goat anti-mouse secondary antibodies (Amersham Biosciences, 1:1,000). After washings, blots were revealed by chemiluminescence assays (ECL, Amersham Biosciences). The resulting signal was detected on Hyperfilm™ ECL (Amersham Biosciences) by using an automated film processor (Hyperprocessor™, Amersham Biosciences).
